# The relationship between emotional neglect and non-suicidal self-injury among middle school students in China: the mediating role of social anxiety symptoms and insomnia

**DOI:** 10.1186/s12888-023-04735-7

**Published:** 2023-04-13

**Authors:** Shiyi Hou, Mireille Twayigira, Xuerong Luo, Lintong Song, Xilong Cui, Qiuxiang Xie, Yanmei Shen, Feilong Yang, Xiuhong Yuan

**Affiliations:** 1 Department of Clinical Psychology, Central South University, The Third Xiangya Hospital, Changsha, 410013 Hunan China; 2 Department of Psychiatry, and National Clinical Research Center for Mental Disorders, The Second Xiangya Hospital of Central South University, Changsha, 410011 Hunan China; 3 Department of General Practice, Central South University, The Third Xiangya Hospital, Changsha, 410013 Hunan China

**Keywords:** Emotional neglect, Non-suicidal self-injury, Social anxiety, Insomnia, Child/adolescent

## Abstract

**Background:**

Non-suicidal self-injury (NSSI) is a vital public concern around the world, and it often starts in adolescence. Emotional neglect (EN) has been considered a distal risk factor for NSSI, but the effects of social anxiety symptoms (SA) and insomnia on this relationship have remained unclear. This study aimed to investigate the potential pathways from EN to NSSI, examining the role of SA and insomnia in this association.

**Methods:**

One thousand three hundred thirty seven Chinese middle school students (M_age_ = 13.040, SD = 0.981, 50.2% males) in China were enrolled in this cross-sectional study. Participants completed the Emotional Neglect sub-scale of Childhood Trauma Questionnaire (CTQ-SF), the Social Anxiety Scale for Adolescent (SAS-A), Athens Insomnia Scale (AIS) and non-suicidal self-injury assessment. Structural equation modelling (SEM) was used to test the possible mediation model among these variables.

**Results:**

231(17.3%) students reported NSSI history during last year and 322 (24.1%) participants reported experiences of EN. Students who experienced EN have higher rates of NSSI compared to students without EN history (29.2% vs 13.5%). EN, SA, insomnia and NSSI were positively related to each other. Furthermore, both SA and insomnia played a mediating role in the relationship between EN and NSSI, the series mediating effect of SA and insomnia on this association was also significant after controlling for demographics. Indirect effects accounted for 58.26% of the total effects (EN → NSSI).

**Conclusions:**

Our study revealed that EN was associated with NSSI, SA and insomnia play indirect roles in the association between EN and NSSI. The findings of our research may have implications for clinicians, families, and schools in their efforts to lower the risk of NSSI in adolescents.

## Background

Non-suicidal self-injury (NSSI), defined as a direct and intentional destruction of body tissue without any observable lethal intent [1], is a significant worldwide mental health concern [2]. The common methods of NSSI include cutting, carving the skin, scratching, banging the head, and so on [1, 3]. In addition to the harmfulness of NSSI itself, prior studies have suggested that NSSI is associated with suicidal ideation, suicide plans, as well as suicide attempts [4–7].

A recent systematic review showed that the lifetime prevalence of NSSI was 17.2% among community adolescents (10–17 years), 13.4% among young adults (18–24 years), and 5.5% among adults (≥ 25 years) [8]. In China, a nationwide survey indicated that about 29% of school-based adolescents in rural China reported a history of NSSI during the past 12 months [9]. With regards to age of onset, findings generally show that NSSI mostly begins between the ages of 12 and 15 [10–13]. For the above reasons, it is necessary to study non-suicidal self-injury behavior among adolescents in order to clarify possible mechanisms of NSSI and provide basis for prevention and intervention.

According to the integrated theoretical model of the development and maintenance of non-suicidal self-injury (NSSI), childhood abuse/ maltreatment is a vital distal risk factor for NSSI [1, 10]. Early adverse experiences (i.e., emotional neglect) may predispose individuals to have high aversive emotions and cognitions, poor distress tolerance, and poor social skills, these individuals may choose NSSI to regulate emotional and social situations when encountering stressful events that result in hyperactivation or inability to cope with [1, 10]. Similarly, the developmental psychopathology perspective establishes an association between childhood trauma and self-injury, a theory suggests that experiences of trauma impair the adaptability of motivational, attitudinal, instrumental, emotional, and relational capacities, which lead to self-injury behavior as a compensatory regulation and relational approach to help individuals meet their development challenges [14]. Emotional neglect is one key type of childhood maltreatment, which refers to “the caregiver failing to meet the child’s basic emotional and psychological needs, including love, belonging, nurturing and support” [15]. Overall, about 18.4% of adolescents (under the age of 18) have reported experiencing emotional neglect as suggested by a meta-analytic review [16]. However, most previous studies have studied the relationships between childhood adversity, general childhood maltreatment or other types of maltreatment (e.g., emotional abuse, physical abuse and sexual abuse) and NSSI; few of them have studied the relationship between emotional neglect and NSSI [17–21]. In addition, in the Chinese cultural context, Chinese parents are less likely to accept and be satisfied with their children; therefore, children are more likely to experience emotional neglect compared to other cultures [22–24]. This indicates the importance of studying the relationship between emotional neglect and NSSI in the Chinese cultural context. Several studies found that emotional neglect may be associated with NSSI, but the results were mixed [9, 25–28] A significant association between EN and NSSI was found in both Kim's and Sandra's studies among college students [25, 26]. In contrast, in a systematic review and meta-analysis published in 2018, all subtypes of childhood abuse were reported to be significantly associated with NSSI, except for the subtype of emotional neglect [28]. Results also differed between two studies conducted in adolescent populations, both of which used the abuse subtype of neglect and did not distinguish between emotional and somatic neglect [9, 27]. One study of 15,623 adolescents in rural China showed a significant association between neglect and NSSI, while another study of 2,038 Canadian adolescents did not find a significant association between neglect and NSSI [9, 27] The confounding of these results may stem from cultural differences in sampling areas, differences in sample age, and whether emotional neglect was chosen as a separate subtype of childhood maltreatment to be studied. Therefore, it is necessary to study the relationship between EN and NSSI in Chinese adolescents. Moreover, most of these studies were conducted to identify the correlates and risk factors of NSSI, while the latent psychological mechanisms that confer risk for NSSI among those who experience emotional neglect from caregivers during childhood are still less understood.

The developmental psychopathology model raised by Yates suggests that childhood traumatic experiences (i.e., emotional neglect) make it difficult for individuals to develop positive expectations of relationships with others and impair the individual's ability to integrate emotional experiences, making it difficult for individuals to form fulfilling relationships with partners, leading these individuals to use self-injurious behavior as a compensatory adjustment [14]. Whereas social anxiety is characterized by a strong fear of evaluation of others in social situations, combined with Yates' theory, emotional neglect is likely to predispose individuals to have higher levels of social anxiety, making individuals more likely to use NSSI to regulate emotions and relationships [14, 29]. Prior research has provided some evidence for the link between social anxiety, emotional neglect and NSSI [30–33]. In addition, studies have suggested that childhood major adverse experiences increase the risk of social anxiety symptoms [30, 31], while in other studies, socially anxious adults have reported greater levels of emotional neglect than healthy controls [32]. However, the participants in most previous studies on this topic have been adults, few studies have focused on adolescents. Coupled with specificities of the psychological and physiological development of the adolescents, and on the other hand the age of onset of social anxiety disorder and NSSI is usually at adolescence, therefore, it is necessary to study the role of SA between EN and NSSI in adolescents [34].

In addition, sleep problems have been associated with higher risk of NSSI. Adolescents’ sleep patterns change due to physiological changes during adolescence and psycho-social factors (schoolwork pressure, parental supervision, class time, electronic device use, etc.) [35–39]. Insomnia is a major sleep problem among adolescents [40]. Based on a developmental psycho-pathological perspective, suffering from insomnia may serve as a developmental challenge for adolescents, due to their poorer motivational, attitudinal, instrumental, emotional, and relational adaptation which related to SA, adolescents who have endured the traumatic experience of emotional neglect may use NSSI as a compensatory strategy to cope with this challenge[14]. In previous studies, adolescents with sleep problems showed a significantly increased NSSI risk [41–43]. Adolescents with sleep problems were significantly had higher odds to report self-harm behaviors than those without sleep problems, reported by a study with large samples [42]. A longitudinal research of Swedish community adolescents sample suggested that poor sleep was a risk factor for NSSI in girls [41]. Likewise, a study conducted among Chinese adolescents (M_age_ = 15.49 years) indicated that poor sleep quality was significantly related to the increased NSSI risk [43]. Besides, previous studies have revealed an association between SA and insomnia [44, 45]. Rodrigo Antunes Lima et al. [45] suggested that the direct and positive correlativity between social anxiety and poor sleep quality has been found both in boys and girls. Similarly, a large sample among American adolescents showed that social anxiety disorder was positively associated with insomnia symptoms [44]. Additionally, studies have demonstrated that childhood maltreatment were associated with the higher risk of sleep disorders in both adolescence and adulthood [46–48] Importantly, as a key type of childhood maltreatment, childhood emotional neglect was found to be the predictor of the higher levels of insomnia symptoms within 2 weeks, after controlling for daily depression among young adults [49]. However, the role of SA symptoms and insomnia in the relationship between emotional neglect and NSSI is still less clear, especially among adolescents.

**Fig. 1:**
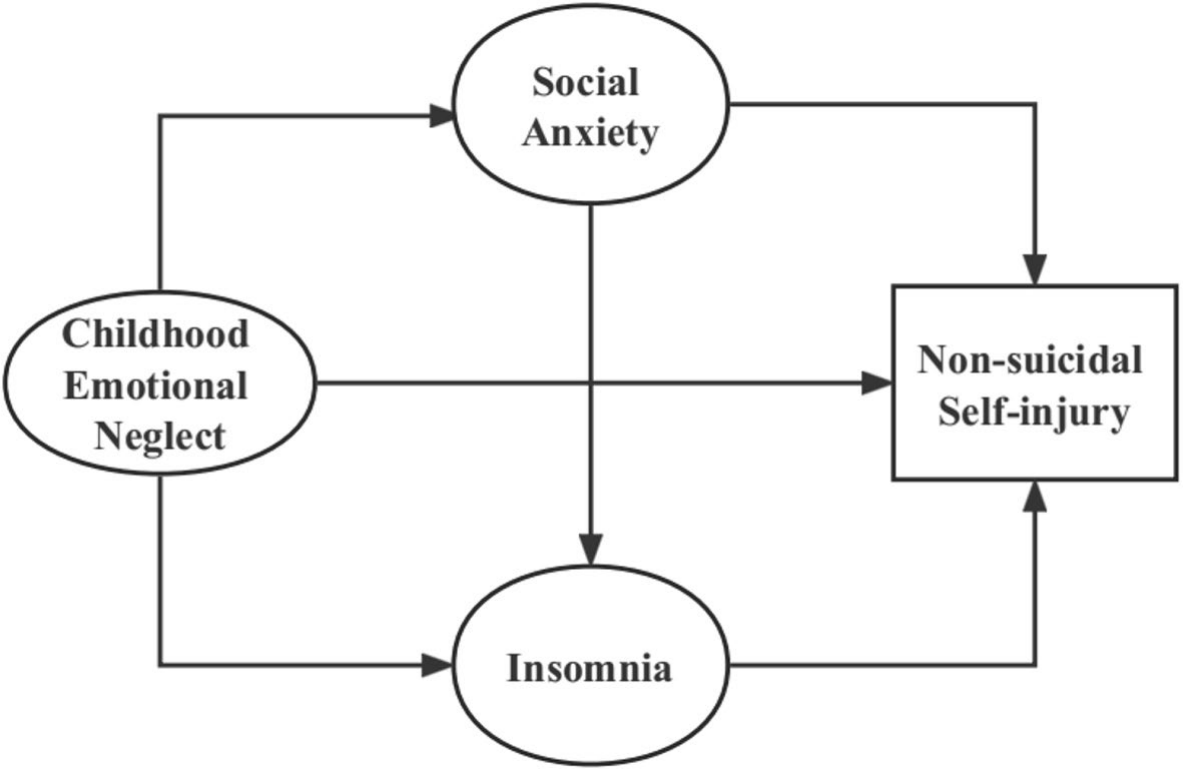
The hypothesized model of the relationship between emotional neglect, social anxiety, insomnia, and non-suicidal self-injury

So far, empirical examination of models linking emotional neglect, social anxiety symptoms, insomnia and non-suicidal self-injury has been limited. In addition, adolescence is a crucial period of transitioning from childhood into adulthood, as well as a sensitive period for the onset of mental health problems [50], especially NSSI [10–13]. A deeper understanding of the relationship between emotional neglect, non-suicidal self-injury, social anxiety symptoms and insomnia among adolescents might shed some light on the mechanisms, prevention, and intervention of these variables. The purpose of this study was to evaluate the relationship between emotional neglect, social anxiety symptoms, insomnia, and non-suicidal self-injury among middle school students, and to examine the role of social anxiety symptoms and insomnia in the relationship between emotional neglect and NSSI. We hypothesized that (1) emotional neglect, social anxiety symptoms, insomnia and non-suicidal self-injury would be associated with each other; (2) social anxiety symptoms and insomnia would play indirect roles in the relationship between emotional neglect and NSSI (social anxiety symptoms and insomnia would mediate the relationship between emotional neglect and NSSI). The hypothetical model is shown in Fig. 1.

## Methods

### Participants and procedures

Before the research began, the present study and its procedure were approved by the Ethics Committee of the Second Xiangya Hospital, Central South University. A cross-sectional design was used in this study. A total of 1610 adolescents aged from 11–16 years were recruited using cluster sampling from a middle school in Changsha, a growing city in central south of China. Surveys were conducted from November 2020 to December 2020. The inclusion criteria for taking part in this study were as follows:(1) middle school students whose age of 11–16 years; (2) participants which were able to read and understand the questionnaires by themselves or by the help of their teacher; (3) without severe physical illness to made them unable to participate; (4) were willing to sign the informed consent forms.

Surveys were delivered via WeChat, a widely used social networking application in China. Before the start of the study, school teachers were systematically trained on how to administer the questionnaire and provide guidance. All students were informed of their right to choose to participant or not and had the right to withdraw at any time. Students and their parents who agreed to participate to this study completed informed consent. 125 students were excluded due to refusal to study and not meeting the inclusion criteria. 148 students who answered regularly and had incomplete data were also excluded, thus 1,337 students (M_age_ = 13.040, SD = 0.981, 50.2% males) were included in final analysis of our study. The valid participation response rate was 83%.

### Measures

#### Emotional neglect sub-scale of childhood trauma questionnaire (CTQ-SF)

Childhood emotional neglect was measured by the sub-scale of the Chinese version of Childhood Trauma Questionnaire (CTQ-SF) [15]. The sub-scale of emotional neglect includes 5 items, which were rated on a 5-point Likert scale ranging from 1 (never true) to 5 (very often true). This scale has shown good reliability and validity in previous studies among Chinese population [51]. Emotional neglect (EN) score ≥ 10 served as the cut-off score in the current study [52]. The Cronbach’s alpha coefficient for the emotional neglect sub-scale was 0.893 in this study.

#### Social anxiety scale for adolescent

The Social Anxiety Scale for Adolescent (SAS-A) [53] was used to assess the levels of social anxiety in the current study. The Chinese version of this scale has shown satisfactory psychometric properties among Chinese population [54]. The Chinese version of this scale is comprised of 13 items, evaluating 3 dimensions: fear of negative evaluations (FNE), social avoidance and distress related to new situations (SAD-N) and social avoidance and distress related to general social contexts (SAD-G). Responses were rated on a 5-point scale ranging from 1 (not at all) to 5 (all the time). The higher the score, the higher the level of social anxiety. The Cronbach’s alpha was 0.949 for SAS-A, 0.939 for FNE, 0.873 for SAD-N, and 0.823 for SAD-G.

#### Athens insomnia scale

The Chinese version of Athens Insomnia Scale (AIS) [55, 56] was administered to evaluate the severity of insomnia in our study. This self-report questionnaire consists of 8 items rated on a 4-point scale (0 to 3: gradually increasing severity), using the diagnostic criteria specified by the International Classification of Diseases (ICD-10). This measurement has shown good validity and reliability in previous studies [55, 57]. The cutoff scores of AIS were as follows: total score ≥ 6 (insomnia), 4 < total score < 6 (suspicious of insomnia), total score < 4 (no insomnia). In the current study, the Cronbach’s alpha of internal consistency reached 0.833.

#### Non-suicidal Self -injury Questionnaire

In the present research, non-suicidal self -injury in the past year was evaluated by two questions which have been widely used in previous studies [58–60]. The first question was “In the past 12 months, have you deliberately hurt yourself without wanting to die (Yes or No)?”, referring to the assessment in the MINI-International Neuropsychiatric Interview for Children and Adolescents 5.0 (MINI KID 5.0). The words “without wanting to die” were bolded and highlighted in red so that participants could pay attention to this expression. If the answer of the first question was “Yes”, the followed second question would be asked as “Have you done these behaviors deliberately to hurt yourself without wanting to die (hitting yourself, pulling your hair, banging your head, pinching yourself, scratching yourself, biting yourself, burning yourself, cutting yourself, or any other ways)”, which refers to Deliberate Self-Harm Inventory (DSHI) [61]. Students who answered positively on both questions were regarded as having NSSI in the past 12 months. Subjects who reported no NSSI behavior history were recorded as 0 points, and those reported a history of NSSI continued to answer the methods in which they took for NSSI, with 1 point for each method, if the subject engaged in 5 types of NSSI, then it was scored 5 (scores range from 0–9 points).

#### Covariates

Gender (male = 1, female = 2), age, grade (7th grade = 1, 8th grade = 2 and 9th grade = 3) and only child (No = 0 or Yes = 1) were included as covariates in present study, because these variables were not only found to be influence factors of NSSI in previous studies [9, 62–65], but also showed significantly correlated with study variables in present study.

### Statistical analysis

The statistical analyses were conducted using SPSS statistical software version 24.0 and the Mplus version 7.0. The common method bias was evaluated using Harman’s single-factor test using SPSS version 24.0. For continuous data, Kolmogorov–Smirnov one-sample test was administered to assess the normality of distribution via SPSS 24.0. Since the scores of EN, SA, insomnia and NSSI were non-normality, Spearman rank correlation was utilized to test the bivariate correlation between the variables. And for categorical variables comparison, χ^2^ test was utilized in this study (gender, grade, nationality, single child and experienced EN or not). When the two-tailed *P* value < 0.05 was considered statistically significant.

Next, the associations between emotional neglect, social anxiety, insomnia and non-suicidal self-injury were assessed by structural equation modeling (SEM) in Mplus 7.0 [66]. For testing the hypothesized model, we performed Anderson and Gerbing’s (1988) [67] two-step strategy in our study. In the first step, confirmatory factor analysis (CFA) was used to test the measurement model. And in the next step, SEM analysis was conducted to measure the coefficients of the pathways and model fit indices of the hypothesized model. The following model fit indices have been suggested by researchers: chi-square (χ^2^) divided by degrees of freedom (χ^2^/df), comparative fit index (CFI), Tucker-Lewis index (TLI), root–mean–square error of approximation (RMSEA), and standardized root-mean-square residual (SRMR). In general, χ^2^/df < 5, CFI > 0.9, TLI > 0.9, RMSEA < 0.08 and SRMR < 0.08 demonstrate that the data and theoretical model fit well [68–70]. We used bootstrapping procedure to test the mediating effects of social anxiety symptoms and insomnia in the relationship between childhood emotional neglect and NSSI, while gender, age, grade (7th,8th and 9th grade) and single child (yes or no) were controlled. When the confidence interval does not include zero, the indirect effect of variables is considered significant.

## Results

### Preliminary analyses

#### Confirmatory factor analysis

Confirmatory factor analysis (CFA) was employed to test the validity of the measurement models before doing the structural equation modeling analysis. The results revealed that the factor loading of each item and sub-scale ranged from 0.490 to 0.935 (all p < 0.001). The measurement models based on previous research fit the data well. In addition, for evaluating the Convergent Validity (CV), the Composite Reliability (CR) and the Average Variance Extracted (AVE) of all constructs were also calculated (see Table 1). The AVE of all constructs ranged from 0.3940 to 0.8225 (AVE > 0.50 indicates that the aggregation validity is good; 0.36 < AVE < 0.50 indicates that the aggregation validity is acceptable), and the CR of all constructs ranged from 0.8358 to 0.9325, exceeding the 0.60 CR threshold value (CR refers to the degree of consistency of a group of items in confirmatory factor analysis). The results showed that the convergent validity was acceptable in our study.

**Table 1:** Confirmatory factor analysis results

Construct	Item	Standardized factor loadings	S.E	*P*-Value	C.R	AVE	CFI	TLI	RMSEA	SRMR
EN	E1	0.677	0.021	***	0.8944	0.6302	0.963	0.926	0.118	0.038
	E2	0.788	0.016	***						
	E3	0.840	0.015	***						
	E4	0.856	0.012	***						
	E5	0.796	0.015	***						
SAS-A	FNE	0.896	0.011	***	0.9328	0.8225	0.941	0.924	0.085	0.05
	SAD-G	0.889	0.013	***						
	SAD-N	0.935	0.012	***						
AIS	A1	0.668	0.022	***	0.8358	0.3940	0.945	0.923	0.067	0.037
	A2	0.490	0.031	***						
	A3	0.509	0.026	***						
	A4	0.768	0.016	***						
	A5	0.723	0.018	***						
	A6	0.570	0.027	***						
	A7	0.632	0.022	***						
	A8	0.608	0.023	***						

(1) *EN* Emotional neglect, *FNE* Fear of negative evaluations, *SAD-G* Social avoidance and distress related to general social contexts, *SAD-N* Social avoidance and distress related to new situations, *SAS-A* Social Anxiety Scale for Adolescent, *AIS* Athens Insomnia Scale. (2) *p < 0.05, **p < 0.01, ***p < 0.001(two-tailed)

### Common method bias test

Exploratory factor analysis was used to test common method bias of the current study. The results showed that the first factor under the unrotated condition accounted for 28.754% of the total variance (< 40%), and KMO = 0.936, Bartlett = 28,490.644, df = 703, *p* < 0.001, indicating that common method bias of this research was not significant.

### Descriptive analyses and correlations

**Fig. 2:**
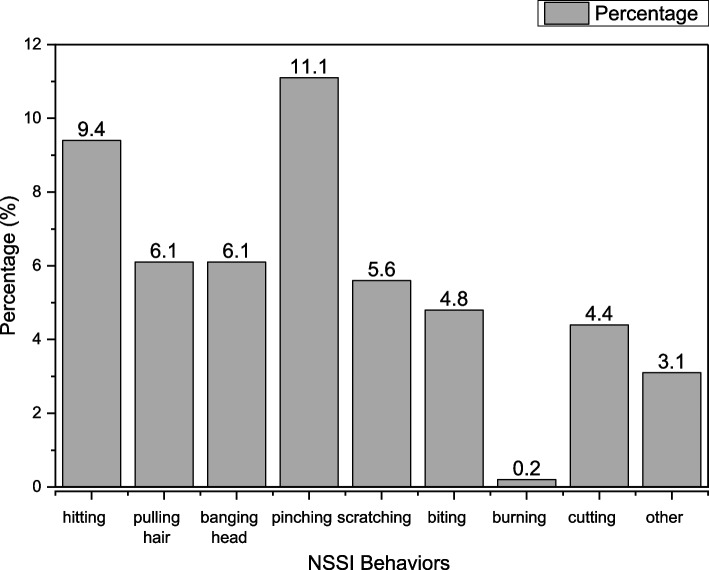
The prevalence of different forms of NSSI behaviors during last 12 months in this sample (*N* = 1337)

Figure 2 shows the 12-month prevalence of different forms of NSSI behavior in this sample. The 12-month prevalence of NSSI was 17.3% (*n* = 231) in this study. Overall, the prevalence of the different forms of NSSI were as follows: self-hitting (9.4%, *n* = 126), pulling hair (6.1%, *n* = 82), banging the head (6.1%, *n* = 82) pinching (11.1%, *n* = 149), self-scratching (5.6%, *n* = 75), biting (4.8%, *n* = 64), self-burning (0.2%, *n* = 3), self-cutting (4.4%, *n* = 59) and others (3.1%, *n* = 41).

Table 2 compared emotional neglect (yes or no) and the demographics between participants with and without NSSI in past 12 months. In total, 24.1% (*n* = 322) of students in this study reported experiencing childhood emotional neglect. Students who experienced emotional neglect have higher rates of NSSI behaviors compared to students without EN history (29.2% vs 13.5%). And participants who reported last 12 months NSSI behaviors were more likely to be girls in our study (*p* < 0.001). However, there were no statistic significant differences between participants with and without NSSI during past 1 year in the variables of grade, nationality and single child.

**Table 2:** Emotional neglect and demographics between participants with and without non-suicidal self-injury during last 12 months

Variable	No NSSI (*n* = 1106)	NSSI (*n* = 231)	χ^2^	df	*P*-value
**Gender**					
Boys	587(43.9%)	84 (6.28%)	21.344	1	< 0.001
Girls	519(38.8%)	147 (11.0%)			
**Grade**					
Grade 7	458(343%)	84 (6.28%)	2.479	3	0.479
Grade 8	326(24.4%)	71 (5.31%)			
Grade 9	321(24.0%)	76 (5.68%)			
**Nationality**					
Han	1050(78.5%)	223 (16.7%)	1.073	1	0.300
Others	56(4.19%)	8 (0.60%)			
**Single Child**					
Yes	470(35.2%)	89 (6.66%)	1.236	1	0.266
No	636 ( 81.7%)	142 (18.3%)			
**EN**					
Yes	228(17.1%)	94 (7%)	42.132	1	< 0.001
No	878(65.7%)	137 (10.2%)			

*NSSI* Non-suicidal self-injury, *EN* Emotional neglect

As regards to the correlation analyses (shown in Table 3), bivariate correlations were conducted to explore the relationships between these variables in our sample of 1337 middle school students (M_age_ = 13.040, SD = 0.981, 50.2% males). The results suggested that all study variables (EN, SA, insomnia and NSSI) were positively and significantly associated with each other (*p* < 0.001, see in Table 3).

**Table 3:** Bivariate Correlations and Descriptive Statistics of Study Variables (*n* = 1337)

	1	2	3	4
1. EN	1.000			
2. SA	0.212^***^	1.000		
3. Insomnia	0.335^***^	0.503^***^	1.000	
4. NSSI	0.226^***^	0.321^***^	0.376^***^	1.000
M	10.620	32.884	4.546	0.509
SD	5.761	13.730	3.804	1.342

*EN* Emotional neglect, *SA* Social anxiety symptoms, *NSSI* Non-suicidal self-injury. *p < 0.05, **p < 0.01, ***p < 0.001(two-tailed)

### Direct and indirect effects

**Fig. 3:**
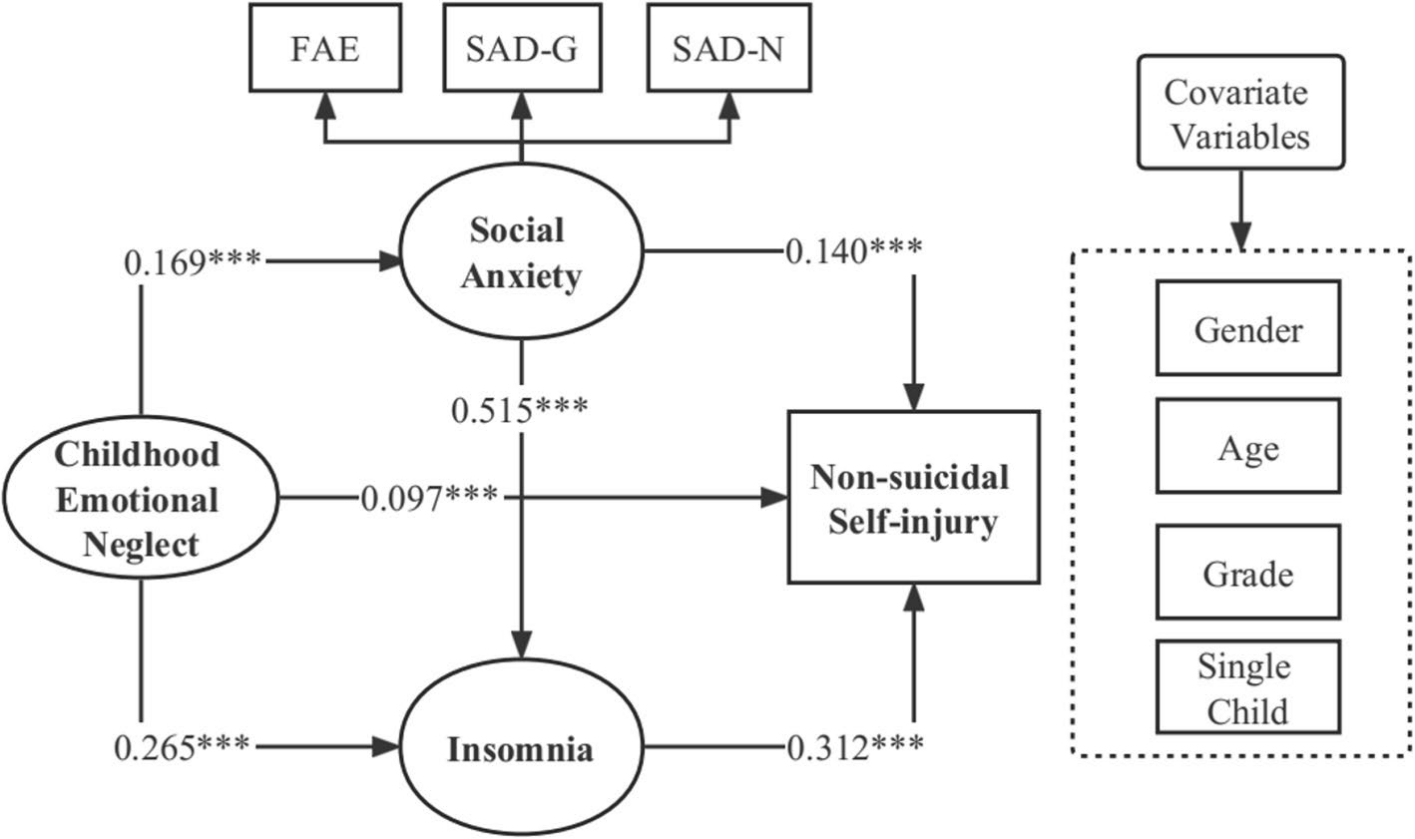
Standardized path coefficients of the direct and indirect relationships between variables Note. (1) *p < .05, **p < .01, ***p < .001(two-tailed); *N* = 1337. (2) χ^2^ = 1770.632, df = 410, χ^2^/df = 4.319, CFI = 0.929, TLI = 0.921, RMSEA = 0.050, SRMR = 0.043

Structural equation modeling (SEM) with Mplus 7.0 was conducted to test the hypothesized model. The results illustrated that the hypothesized model fit the data well (χ^2^ = 1770.632, df = 410, χ^2^/df = 4.319, CFI = 0.929, TLI = 0.921, RMSEA = 0.050, SRMR = 0.043). Figure 3 shows the standardized coefficients for all paths in the model after controlling for gender, age, grade and only child. The paths from emotional neglect (β = 0.097, *p* < 0.001), social anxiety (β = 0.140, *p* < 0.001) and insomnia (β = 0.312, *p* < 0.001) to non-suicidal self-injury were positive and significant. Similarly, the paths from childhood emotional neglect (β = 0.265, *p* < 0.001) and social anxiety (β = 0.515, *p* < 0.001) to insomnia were also significant. Additionally, emotional neglect was positively and significantly related to social anxiety (β = 0.169, *p* < 0.001).

For non-normality distribution of EN, SA, insomnia and NSSI, according to Tofighi’s suggestion [71], we used percentile bootstrapping at a 95% confidence interval with 5,000 bootstrap samples in order to verify if the hypothesized mediating effects of social anxiety and insomnia were present in the current study.

As shown in Table 4, the results of the bootstrap test confirmed the existence of a positive and significant mediating effect of social anxiety in the relationship between emotional neglect and non-suicidal self-injury (indirect effect = 0.033, *p* = 0.002, 95% CI [0.015, 0.058]), a mediating effect of insomnia in the relationship between emotional neglect and non-suicidal self-injury (indirect effect = 0.117, *p* < 0.001, 95% CI [0.075, 0.164]), and positive and significant distant mediating effects of social anxiety and insomnia between emotional neglect and non-suicidal self-injury (indirect effect = 0.038, *p* < 0.001, 95% CI [0.020, 0.062]). The 95% confidence interval of all paths did not include 0, indicating that the mediating effects of social anxiety symptoms and insomnia were significant, and the chain mediating effect of social anxiety symptoms and insomnia was also significant. Total indirect effects accounted for 58.26% of the total effects, while the indirect effect 1 (emotional neglect → social anxiety symptoms → NSSI), indirect effect 2 (emotional neglect → insomnia → NSSI) and indirect effect 3 emotional neglect → social anxiety → insomnia → NSSI) accounted for 10.43%, 36.09% and 11.74% respectively. Then, we compared the indirect effect sizes of the three pathways. The indirect effect of EN through of SA affecting NSSI was significantly smaller than which through insomnia (*p* = 0.002), while the effect of insomnia in the relationship between EN and NSSI was significantly greater than the distant mediating effect of SA and insomnia between EN and NSSI(p < 0.001). Additionally, the indirect effect size of pathway “EN → SA → NSSI” was not statistically different from the indirect effect size of pathway “EN → SA → insomnia → NSSI”. Detailed statistics are reported in Table 4.

**Table 4:** Direct, indirect, and total effects of the hypothesized model after controlling for demographics

Path	Point Estimate	Product of Coefficients			Bootstrap 5000 Times 95% CI		Effect Size
		S.E	Z	*P*-Value	Lower	Upper	
INDIRECT EFFECTS							
EN → SA → NSSI	0.033	0.011	3.046	0.002	0.015	0.058	10.43%
EN → Insomnia → NSSI	0.117	0.023	5.091	< 0.001	0.075	0.164	36.09%
EN → SA → Insomnia → NSSI	0.038	0.011	3.638	< 0.001	0.020	0.062	11.74%
Total Indirect Effect	0.188	0.032	5.968	< 0.001	0.132	0.255	58.26%
DIRECT EFFECTS							
EN → NSSI	0.136	0.039	3.502	< 0.001	0.060	0.213	42.17%
TOTAL EFFECT							
Total Effect	0.325	0.049	6.613	< 0.001	0.231	0.425	100.00%
CONTRASTS							
SA VS. Insomnia	-0.083	0.027	-3.120	0.002	-0.138	-0.033	
SA VS. SA → Insomnia	-0.005	0.013	-0.384	0.701	-0.032	0.019	
Insomnia VS. SA → Insomnia	0.078	0.020	3.928	< 0.001	0.042	0.120	

Non-standardized estimating of 5000 bootstrap sample; The ratio of effects to total effect were calculated using standardized coefficients, *EN* Emotional neglect, *SA* Social anxiety symptoms, *NSSI* Non-suicidal self-injury

## Discussion

Our findings indicate that both NSSI and childhood emotional neglect (EN) are prevalent among Chinese middle school students, which also emphasizes the importance of focusing on the relationship between NSSI and childhood emotional neglect. The possible underlying mediating mechanism of this association (how social anxiety symptoms and insomnia influenced the association of EN and NSSI) were examined in this sample of middle school students in China. As hypothesized, emotional neglect, social anxiety symptoms, insomnia and non-suicidal self-injury were positively related to each other after controlling for age and gender. Moreover, social anxiety symptoms and insomnia significantly mediated the relationship between EN and NSSI, which may reveal the underlying mechanism of this association. Next, each of our results will be discussed below.

The prevalence of non-suicidal self-injury among adolescents in past 12 months was 17.3% in our study, similar to a previous study [8]. Inconsistent with our observation, a study conducted in rural China reported that about 29% of the participants had NSSI history during last 1 year, which is much higher than the rate found in our study [9]. However, our study only included middle school participants, while this study was performed among both middle school students and high school students, which may lead to a higher prevalence. Secondly, the discrepancy may also be due to the different sociodemographic characteristics, cultures, environments and what urban and rural adolescents have experienced in China. Furthermore, the difference in sampling area may also contribute to the variation. Moreover, the top three forms of NSSI behavior found in our study were pinching, hitting and pulling hair, basically consistent with a previous study in China [9], and this may stem from the common Chinese context.

Additionally, 24.1% of the participants reported a history of emotional neglect in the present study, which is within the range of a previous review, but above the global self-reported rate [16]. This finding may be related to the characteristics of Parenting styles in China and the differences with other cultures, which further underscores the importance of paying attention to the emotional neglect experienced by adolescents [22–24].

In line with prior studies, we found that emotional neglect was positively associated with NSSI behavior [9, 25, 26]. In support of the integrated theoretical model of NSSI [1, 10], emotional neglect is one of the distal risk factors of NSSI which may contribute to an individual’s intrapersonal and interpersonal vulnerabilities, which may lead to responding to stressful events in a maladaptive way (NSSI may serve as a maladaptive stress-coping strategy), leading to an increased risk of NSSI.

Our study moved beyond the findings of existing research and provided the evidence for the indirect effects of social anxiety symptoms and insomnia in the relationship between emotional neglect and NSSI. Social anxiety symptoms were found to significantly mediate the association between childhood emotional neglect and NSSI in the current study. Consistent with the integrated theory of NSSI [1, 10], emotional neglect history could lead to problems with affect regulation and interpersonal communication while social anxiety has been found to be related with emotional dysregulation in prior studies [72, 73], and this could increase the risk of NSSI, considering that NSSI is a maladaptive way used to immediately regulate and relieve negative emotions in negative experiences or unpleasant social interactions. Previous research has also shown that emotional neglect is associated with social anxiety, which is also in line with our research results [30–33]. Our study has also showed a possible link between social anxiety and NSSI, which extends the research findings on the relationship between anxiety and NSSI [7, 59, 74, 75].

Additionally, emotional neglect also affected NSSI through insomnia in our study. Adolescents with more emotional neglect experiences exhibited higher levels of insomnia, and this may have contributed to increased likelihood of engagement in NSSI. A previous study has supported the assertion that emotional neglect is related to insomnia symptoms [49], and similarly, several studies have shown a positive association between sleep problems and NSSI in adolescents [41–43]. Our results have provided evidence for the underlying relationship between childhood emotional neglect, insomnia and NSSI based on existing research, and a reference for future studies on the relationship between these three variables.

Furthermore, a series effect of social anxiety symptoms and insomnia mediating the association between emotional neglect and NSSI was found in our study, helping to understand the mechanism of the relationship between EN and NSSI more deeply. Our findings have further proved that social anxiety may be associated with sleep disorders, which is in accordance with the results of previous studies [44, 45, 76]. Implicit high vigilance for social threats may reduce sleep quality [77], which may explain the mechanism of the association between social anxiety symptoms and insomnia. The series mediating effect of social anxiety symptoms and insomnia demonstrates that adolescents with higher levels of emotional neglect might be more likely to experience higher levels of social anxiety due to poor emotion regulation and social contact [72, 73], and in turn, social anxiety symptoms may impair the sleep quality of the adolescents [44, 45, 76, 77], further increasing the risk of NSSI behavior. Our research has shown that childhood emotional neglect may not only have a direct effect on the occurrence of NSSI but may also affect NSSI behavior through the indirect effects of social anxiety symptoms and insomnia.

Several limitations need to be considered in the current study. Firstly, this study used cross-sectional design to examine the hypothesis model; therefore, causality in the relationship between emotional neglect, social anxiety symptoms, insomnia and NSSI cannot be deduced; longitudinal data should be used to further verify the relationship between these variables. Secondly, the participants were recruited from a specific region of China, so the findings cannot be directly generalized to adolescents in other regions. Thirdly, self-reports are prone to social desirability bias and recall bias. However, some psychological factors are not easily observed; therefore, self-reporting questionnaires are commonly used in research. Multiple methods could be included in future studies, such as interviews, observation, and parents-report questionnaires. Fourthly, some possible factors were not included in the present study. Depression, generalized anxiety and other types of childhood maltreatment may be associated with CEN, SA, insomnia and NSSI, and these variables should be evaluated and controlled for in future studies. In addition, the assessment of NSSI used seven common behaviors, which have been used in prior studies [58–60]; it is significant to use a measurement of more validity such as Self-Injurious Thoughts and Behaviors Interview [78]. Moreover, current study did not consider the effect of left-behind experience, which is one of the most vital causes of emotional neglect in China and is also a risk factor for NSSI, needs to be considered for further research [79–81]. Finally, the links between emotional neglect, social anxiety symptoms, insomnia and NSSI might be bidirectional. In future studies, the different directionalities in the relationships between these variables could be tested.

Despite these limitations, the results of the current study have some implications for clinical practices and research. Firstly, we evaluated the connections between emotional neglect, social anxiety symptoms, insomnia and NSSI based on the hypothesis model, providing some evidence for a deeper understanding of NSSI developing mechanism. Secondly, our research findings emphasized the impact of emotional neglect, which has great implications for both clinical and family education. In clinical prevention and intervention of NSSI, social anxiety disorder and insomnia, it may be pivotal to take into account the role of emotional neglect. In addition, our findings offer a lesson for parents; less emotional neglect and more care is an effective way to make their children mentally healthier. Thirdly, the indirect effects of social anxiety and insomnia on the association between emotional neglect and NSSI were also highlighted in our study. These pathways suggest that lessening the symptoms of social anxiety as well as insomnia may conduce to the decrease of NSSI risk. In addition, we should also attach an importance to verifying whether these results are applicable to clinical samples in future studies.

## Conclusions

Taken together, our work reveals possible links between emotional neglect, social anxiety symptoms, insomnia and NSSI, using a sample of middle school students in China. Childhood emotional neglect is associated with social anxiety symptoms, insomnia and NSSI. Social anxiety symptoms are associated with insomnia and mediate the relationship between childhood emotional neglect and NSSI. Childhood emotional neglect also affects NSSI through insomnia. And to take it further, our study uncovers that social anxiety symptoms and insomnia have a chain mediating effect on the association between childhood emotional neglect and NSSI. Our findings may have important implications for clinical treatment, family, and school education in order to lower the risk of NSSI in adolescents.

## Data Availability

All transcript fragments for which analysis is provided in this publication are included in the paper and its supporting information. The full texts generated in the current study are not publicly available as they may identify individuals, even if they are anonymous, but are available from the corresponding author upon reasonable request.

## Abbreviations

NSSI: Non-suicidal self-injury
EN: Emotional neglect
SA: Social anxiety symptoms
SAS-A: Social anxiety scale for adolescent
AIS: Athens insomnia scale
SEM: Structural equation modelling
CTQ-SF: Chinese version of childhood trauma questionnaire
FNE: Fear of negative evaluations
SAD-N: Social avoidance and distress related to new situations
SAD-G: Social avoidance and distress related to general social contexts
ICD-10: International classification of diseases
MINI KID 5.0: MINI-international neuropsychiatric interview for children and adolescents 5.0
DSHI: Deliberate self-harm inventory
CFI: Comparative fit index
TLI: Tucker-Lewis index
RMSEA: Root–mean–square error of approximation
SRMR: Standardized root-mean-square residual
SD: Standard deviation
CFA: Vonfirmatory factor analysis
CV: Convergent validity
CR: Composite reliability
AVE: Average variance extracted
CI: Confidence interval
BCCI: Bias corrected confidence interval
SE: Standard error
